# Development and Evaluation of a Novel Sulfonated Phenol–Formaldehyde Resin with High Dispersion Stability

**DOI:** 10.3390/polym14214682

**Published:** 2022-11-02

**Authors:** Xianxing Meng, Likun Wang, Hongwei Wang, Fang Zhang, Taying Su, Kunmu Cheng

**Affiliations:** 1 School of Chemistry and Chemical Engineering, Ankang University, Ankang 725000, China; 2 Ankang Research Centre of New Nano-Materials Science and Technology, Ankang University, Ankang 725000, China; 3 China University of Petroleum (Beijing), Beijing 102249, China; 4 Jilin Oilfield Development Department, Songyuan 138001, China; 5 Xinjiang Karamay Caifeng Industrial Co., Ltd., Karamay 834000, China

**Keywords:** phenol–formaldehyde resin, sulfonation, characterization, HPLC–MS/MS

## Abstract

Sulfonated phenol–formaldehyde (SPF) resin used as a cross-linker for petroleum reservoir conformance control was synthesized under alkaline conditions. The reaction process of SPF resin was evaluated by measuring the solution’s viscosity with respect to phenol–formaldehyde (PF) resin. The molecular structure of SPF resin was characterized by both FTIR and HPLC–MS/MS. The influence of the formaldehyde/phenol molar ratio (F/P) and the sodium formaldehyde sulfoxylate/phenol molar ratio (S/P) on the properties of SPF were analyzed in terms of the storage time, coagulation value, molecular size, and zeta potential. The results indicate that the presence of formaldehyde sodium bisulfite could slow down condensation reaction. Phenol rings were connected by methylene bridges in the position of o–p, and sulfonated SPF resin molecules all had one sulfonate group on the oligomer structure. The storage time decreased from 87 to 6 days, and the zeta potential decreased from −3.02 to −7.70 mV with the increase in F/P (1.2–2.0). Meanwhile, the sedimentation value and the diameter increased from 3.291 × 10^4^ to 5.045 × 10^4^ mg/L and from 2.7 to 5.3 nm, respectively. Sulfonation could significantly increase the storage time and dispersion stability. With the increase in S/P (0.1–0.35), the storage time increased from 15 to 86 days, the sedimentation value increased from 1.927 × 10^4^ to 5.269 × 10^4^ mg/L, and the diameter decreased from 6.3 to 3.0 nm. This paper can present new ideas for improving the storage stability and salt tolerance of phenol–formaldehyde resin and further improving the range of its applications, which has essential reference significance.

## 1. Introduction

PF resin, invented by Baekelana in 1909, is a polycondensation of phenols and aldehydes catalyzed by acids or bases at certain temperatures [1] It is the first industrially produced polymer material, and due to its low cost, heat and chemical resistance, flame-retardant capabilities, and other advantages, it is widely used in the production of thermosetting resins, coatings, adhesives, etc [2]. At present, it has become an indispensable polymer material in the fields of aerospace, building materials, and electronic device manufacturing [3]. 

Phenol and formaldehyde are the two most commonly used materials in the synthesis of phenolic resin [4]. By controlling the molar ratio of phenol to formaldehyde, the reaction temperature, and the reaction time under alkaline conditions, polyhydroxymethyl PF resin was obtained [5]. PF resin has certain high dispersion stability under alkaline conditions and is a kind of environmental cross-linker widely used for the profile control [6,7]. The hydroxymethyl group on the molecular structure can cross-link with the amide group on the polyacrylamide to form a network gel system under reservoir conditions, which is widely used in the profile control and plugging of middle-to-high-temperature oil field gel [8,9].

The synthesis of PF resin is affected by the molar ratio of the reaction material, the type of catalyst, the amount of catalyst, the reaction time, the reaction temperature, the way of adding formaldehyde, and other factors, which determine the structure and properties of the resin [10]. In the process of PF resin synthesis, the commonly used alkaline catalysts include sodium hydroxide, barium hydroxide, ammonia, magnesium hydroxide, calcium hydroxide, and tertiary amine [11]. The dosage of sodium hydroxide was 1%~5%, the dosage of barium hydroxide was 3%~6%, and the dosage of hexamethyltetramine was 6%~12% [10]. The presence of sodium and potassium ions, as well as higher pH conditions, is favorable for para-substitution. However, calcium, barium, and magnesium ions and low pH values are favorable for ortho-substitution [12]. Ammonia catalysts can directly participate in the resin reaction, and the obtained resin has a relatively high molecular weight but poor water solubility [13]. The use of a strong base catalyst can significantly increase the content of hydroxylmethyl and the solubility of the resin in water [14]. G. Astarloa Aierbe et al. [13] investigated the effect of the phenolic molar ratio on the reaction kinetics of five thermosetting PF resin prepolymers. It was found that the maximum concentration of the reaction products decreased with the increase in the phenolic molar ratio, but the maximum concentration of the double and trisubstituted phenol was independent of the phenolic molar ratio. When the molar ratio of phenol and formaldehyde of thermosetting phenolic resin is less than 1, and the pH value is less than 3, formaldehyde can undergo an electrophilic substitution reaction and form hydroxymethyl with phenol. The resulting hydroxymethyl continues to react with other phenols to further increase the chain and finally obtain thermoplastic phenolic resin [15]. When the pH value of the reaction solution is between 3.0 and 3.1, the mixture of phenol and formaldehyde cannot react, even by boiling it for a long time. This range is also called the neutral point of the reaction of phenolic resin. When the pH value of the reaction solution is between 7.0 and 11.0, phenol can generate phenolic oxygen-negative ions under alkaline conditions, which can react with formaldehyde to form hydroxymethyl phenol, and the further reaction of hydroxymethyl phenol can obtain thermosetting phenolic resin [16]. 

However, the dispersion stability of PF resin cross-linker products is easily affected by the ionic strength, and the hydroxylmethyl grouphas high reactivity, which results in self-polymerization. The poor solubility in reservoir injection water is prone to molecular aggregation and the formation of aggregates, and the field storage period is short, which affects the extent and effect of its field application. Sulfomethylated phenolic resin was obtained by introducing sodium formaldehyde sulfoxylate in an alkaline condition. The reaction mechanism of sulfonated phenolic resin was analyzed by comparing the changes in the viscosity of the sulfonated phenolic resin and phenolic resin. The molecular structure of SPF resin was characterized using FTIR and HPLC–MS/MS. Therefore, the effects of the formaldehyde/phenol molar ratio (F/P) and the sodium formalhyposulfonate/phenol molar ratio (S/P) on SPF performance were analyzed in terms of the storage time, coagulation value, molecular size, and zeta potential.

## 2. Materials and Methods

### 2.1. Materials

Phenol (99.9%) and formaldehyde (37% aqueous solution) were analytical reagents used without further purification purchased from Shanghai Aladdin Biochemical Technology Co., LTD, Shanghai, China. Sodium formaldehyde sulfoxylate was synthesized in our laboratory. Deionized water was used for the preparation of all aqueous solutions. Other chemicals such as sodium hydroxide and sodium hydrogen sulfite were purchased from Enokai Co., LTD, Beijing, China.

#### 2.1.1. Preparation of Sodium Formaldehyde Sulfoxylate

Deionized water and sodium hydrogen sulfite were mixed in a weight ratio of 1:1. After fully mixed, the sodium hydrogen sulfite solution was transferred to a three-neck flask. Then, 37 wt% formaldehyde, which has the same molar amount as sodium hydrogen sulfite, was added to the three-neck flask through a constant pressure funnel when the mixture was heated to 50 °C. After the addition of formaldehyde, the mixture was reacted at 50 °C for 4 h.

#### 2.1.2. Preparation of SPF Resin

The SPF resin was prepared in a three-neck flask, which was equipped with a mechanical stirrer, a cooling condenser, a constant pressure funnel, and a water bath with a certain amount of phenol, formaldehyde, and sodium formaldehyde sulfoxylate. Phenol was preheated at 50 °C for liquidation. Phenol, formaldehyde, and sodium formaldehyde sulfoxylate were mixed in the three-neck flask. Briefly, a 40 wt% sodium hydroxide solution was added to the mixture until the pH reached a value of 9.3–9.5. In the first step, the mixture was heated for 45 min at 80 °C. Then, the mixture was reacted at 90 °C for 120 min. After this, the flask was cooled to room temperature to stop the reaction. PF was synthesized in the same way as SPF, except that no sodium formaldehyde sulfoxylate was added to the reaction solution.

### 2.2. Methodology

#### 2.2.1. FTIR

FTIR analysis was carried out using a MAGNA-IR 560ESP spectrometer (Thermo Fisher Nicolet, MA, USA) in the range of 400–4000 cm^−1^ with a resolution of 0.35 cm^−1^. The SPF resin sample was freeze-dried at −50 °C to obtain powder samples before characterization. When IR radiation passed through a dry sample, different groups selectively absorbed infrared light of different wavelengths.

#### 2.2.2. HPLC–MS/MS

The HPLC–MS/MS system consisted of an HPLC system (Agilent Technologies, Palo Alto, CA, USA) composed of a quaternary pump, a degasser, and an auto-sampler. The model of mass spectrometry (MS) was an LCQ DecaXP (Thermo Finnigan) quadrupole ion trap tandem mass spectrometer with an ESI interface operating in the negative ion mode. Three abundant ions in the MS scan were evaluated by MS/MS scans to further obtain information about the SPF resin’s molecular structure.

The following gradient system was used with water (solvent A) and acetonitrile (solvent B): 0 min, 5% B; 5 min, 5% B; 20 min, 20% B; 30 min, 70% B; 40 min, 90% B; and 50 min, 90% B, followed by washing and re-equilibrating the column. The column temperature was 25 °C, and the flow rate was maintained at 0.2 mL/min.

The effluent from the HPLC column was directed into the electrospray source of MS, which was operated in the negative ion mode. Nitrogen gas, which was required for the MS, was generated using an in-house nitrogen generator. The ESI conditions were as follows: ion range, m/z 150–1800; capillary voltage, 3.5 kV; nebulizer gas pressure, 37.5 psi; drying gas flow, 1.05 mL/min; and desolvation temperature, 275 °C. The MS/MS analysis was performed by using helium as the collision gas at a pressure of 4.6 × 10^−6^ mbar.

#### 2.2.3. Viscosity

**Figure 1:**
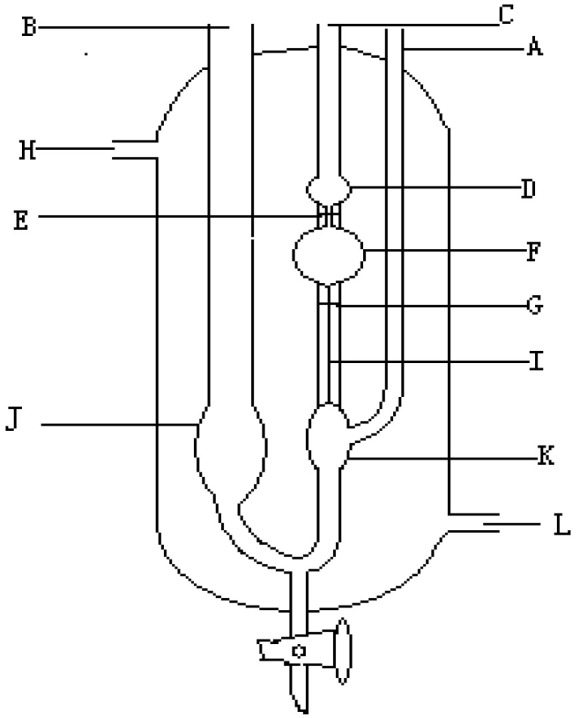
Ubbelohde viscometer structure: (A) air tube, (B) injection tube, (C) pipette, (D) buffering sphere, (E) upper tick mark, (F) quantify sphere, (G) lower tick mark, (I) capillary, (L) water inlet, (H) water outlet tube, (J) tube reservoir and (K) hanging horizontal reservoir.

Since the viscosity of PF or SPF resin solutions is related to their relative molecular weight and the extent of polycondensation, an Ubbelohde viscometer was used to determine the reaction product viscosity [17]. The viscometric conditions were as follows: PF or SPF resin solutions were formulated to a mass concentration of 1000 mg/L using deionization and measured at 25 °C. Figure 1 shows the Ubbelohde viscometer structure.

#### 2.2.4. Storage Time

During storage, the SPF resin product can experience a slow condensation reaction. This causes an increase in its viscosity, and finally, SPF resin loses flowability, which greatly restricts its application. The storage time was used to evaluate the stability of SPF resin during storage. Briefly, 5 mL of an SPF solution (30 wt%) was placed in a 10 mL test tube and sealed. After that, it was placed in a 40 °C oven. The test tube was inverted every day for observation, and the time was recorded when all the solutions could not flow down in one minute, which was the storage time.

#### 2.2.5. Coagulation Value 

The coagulation value was used to evaluate the salt resistance of SPF resin. A certain amount of 1000 mg/L SPF resin solution prepared with deionized water was put in a conical flask. Then, the SPF resin solution was titrated using a sodium chloride solution with a known concentration until the solution become turbid, and the volume of the used sodium chloride solution was recorded. The calculation formula of the coagulation value is shown as follows:

$$\mathrm{Coagulation}\;\mathrm{value}=\frac{c_{1}V_{1}}{V_{1}{+V}_{2}}$$

where *c*_1_ is the concentration of the sodium chloride solution; *V*_1_ is the volume of the used sodium chloride solution; *V*_2_ is the volume of the titrated SPF resin solution.

#### 2.2.6. Molecular Size and Zeta Potential

The molecular size and zeta potential of the synthesized SPF resin were measured using a particle analyzer (Delsa Nano C, Beckman Coulter, Fullerton, CA, USA). A scattering angle of 150° was applied to measure the SPF resin size. The autocorrelation function of the CONTIN algorithm was used for calculating the SPF resin molecular size. The zeta potential was determined via the electrophoretic light scattering technique and was calculated from electrophoretic mobility using the Stokes–Einstein equation:

$$d=\frac{kT}{3\pi \eta D}$$

where *d* is hydrodynamic diameter, *D* is the translational diffusion coefficient, *k* is Boltzmann’s constant, *T* is the absolute temperature, and *η* is the viscosity.

## 3. Results

### 3.1. Analysis of Reaction Process for SPF Resin

The reaction of SPF resin includes additive reaction and condensation reaction. The additive reaction is the addition of formaldehyde and sodium formaldehyde sulfoxylate to ortho- and para-phenol positions. Previous results [18] have shown that the additive reaction mainly results in monofunctionalized derivatives. The condensation reaction primarily occurs between phenol-free positions and hydroxymethyl groups, producing a methylene bridge leading to a higher molecular weight [19]. Because the viscosity of the SPF resin solution can be related to the resin’s molecular weight, the viscosity of the SPF resin solution can provide information about the degree of the condensation reaction. In this section, the SPF resin reaction process is evaluated by measuring the solution viscosity and comparing it to that of PF resin. 

**Figure 2:**
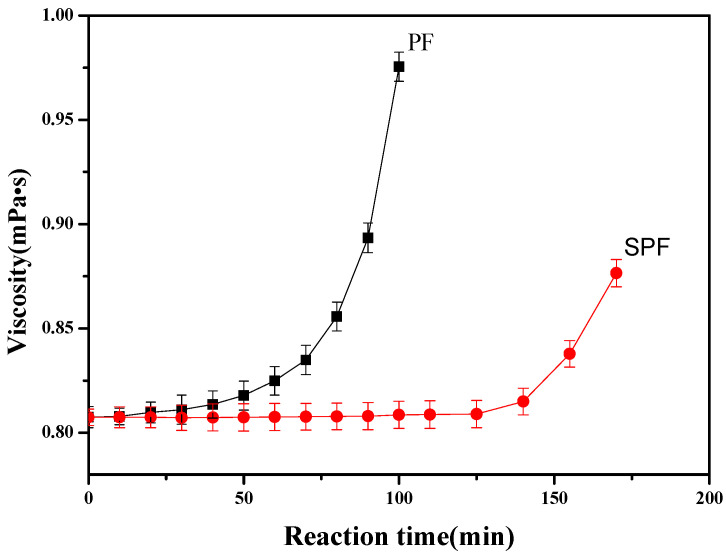
Viscosity of SPF resin compared with that of PF resin (PF resin: F/P was 2.0, and S/P was 0; SPF resin: F/P was 2.0, and S/P was 0.2; mean ± s.d., *n* = 3 per condition).

The viscosity evaluation of the SPF resin solution (1000 mg/L) is shown in Figure 2, in which it is compared with a PF resin sample that had the same phenol/formaldehyde molar ratio and other reaction conditions. It is observed that when the reaction time was smaller than 60 min, little increase was obtained in the viscosity of PF resin (curve A). When the reaction time was longer than 60 min, the viscosity of the PF resin solution quickly increased, and the reaction product lost flowability when the reaction time was longer than 100 min. This result indicates that, when the reaction time was smaller than 60 min, the additive action in PF resin was in the dominant position, resulting in a slow increase in the viscosity of PF resin. When the reaction time was longer than 60 min, the condensation reaction overwhelmed the additive reaction, leading to a fast increase in the PF resin’s viscosity. However, for the SPF resin, the solution viscosity remained nearly constant for 125 min, and after that, the viscosity noticeably increased with the reaction time. When the reaction time was longer than 150 min, the reaction product lost flowability, which was much longer than that of PF resin. This result demonstrates that the addition of sodium formaldehyde sulfoxylate can effectively decrease the condensation reaction of SPF resin, compared with PF resin. 

### 3.2. Structure Analysis

#### 3.2.1. FTIR

**Figure 3:**
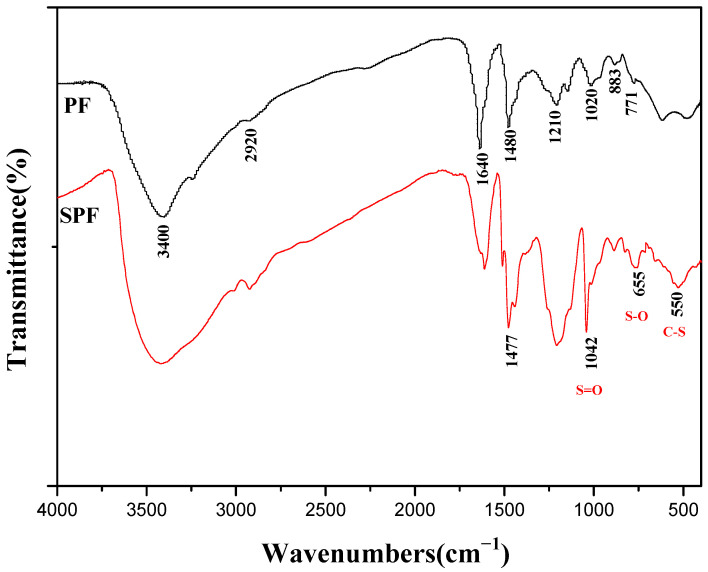
FTIR spectra of SPF and PF resin samples (PF resin: F/P was 2.0, and S/P was 0; SPF resin: F/P was 2.0, and S/P was 0.2).

Figure 3 shows the FTIR spectra of SPF resin and PF resin. Both SPF and PF resin showed adsorption peaks at 771 and 883 cm^−1^, corresponding to the out-of-plane vibration of C-H on the aromatic nucleus [9]. The adsorption at 1020 cm^−1^ was assigned to the vibration of primary hydroxyl groups (C-O) [20]. The wide band at 3400 cm^−1^ was related to the O-H stretching vibration of the phenolic hydroxyl group, while the adsorption peak at 2920 cm^−1^ was attributed to C-H stretching in methyl or methylene groups. The adsorption at 1640 cm^−1^ was mainly attributed to carbonyl stretching, which resulted from the oxidation of the phenolic hydroxyl group. The adsorption peak at 1210 cm^−1^ was attributed to the Ph-O stretching vibration of the phenolic hydroxyl group. Furthermore, the adsorption peaks of the FTIR spectrum between 1400 and 1500 cm^−1^ have information about the structure of methylene bridges, according to Roczniak’s study [21]. The adsorption peak at about 1450 cm^−1^ was related to methylene bridges in p–p′, the band at approximately 1460 cm^−1^ was assigned to the methylene bridge in o–o′, and the adsorption at 1480 cm^−1^ contributed to the o–p′ position. The two adsorptions at about 1480 cm^−1^ indicate that phenol rings were connected by methylene bridges in the position of o–p′ for the two resin samples.

A new absorption value appeared in the SPF resin at 550 cm^−1^, mainly attributed to the C-S stretching vibration, and the adsorption at 650 cm^−1^ was attributed to the S-O stretching vibration of sulfonic group [22]. Moreover, the adsorption at 1042 cm^−1^ in the spectrum of the SPF resin was the characteristic absorption of sulfonic groups [23]. These new adsorption values for the SPF resin compared with the PF resin show that the SPF resin was successfully sulfonated with -CH_2_SO_3_Na groups.

#### 3.2.2. HPLC–MS/MS Technology

**Figure 4:**
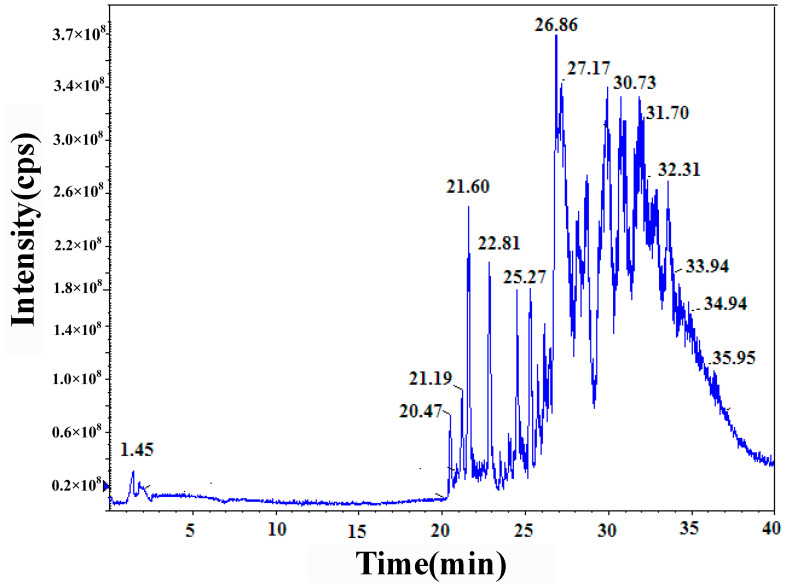
Total ion current chromatogram of SPF resin product (F/P was 2.0, S/P was 0.2).

To determine the SPF resin’s molecular structure, HPLC–MS/MS was used with electrospray ionization under a negative ion condition. Figure 4 shows the total ion current chromatogram of the SPF product. It is clearly seen that the chromatographic peaks were well-separated in the retention time (below 30 min). However, when the retention time exceeded, the chromatographic peaks overlapped. According to this total ion current chromatogram, the mass spectrums of the samples were analyzed with different retention times. The mass spectrum data in different retention times are shown in Table 1. Only deprotonated molecule [M-H]^−^ ions (m/z 319.5, 425.6, 455.4, 531.6, 561.5, and 591.5) could be identified with the retention time in the range of 21–26 min. Peak-to-peak mass increments include 30, 106, and 136 Da, which were repeating units in phenol–formaldehyde resin. In the retention time of 31.7–32.7 min, only [M-Na]^−^ ions were found, in which the molecular structures were all found to have one sulfonate group, as shown in Table 1. The peak-to-peak mass increments were 30, 106, and 136 Da, which represent the liberating methylol and phenolic ring groups. With higher retention time in the range of 34.7–35.0 min, the [M-H]^−^ and [M-Na]^−^ ions were all identified. As shown in Table 1, [M-Na]^−^ ions all had one -CH_2_SO_3_Na group on the oligomer structure.

**Figure 5:**
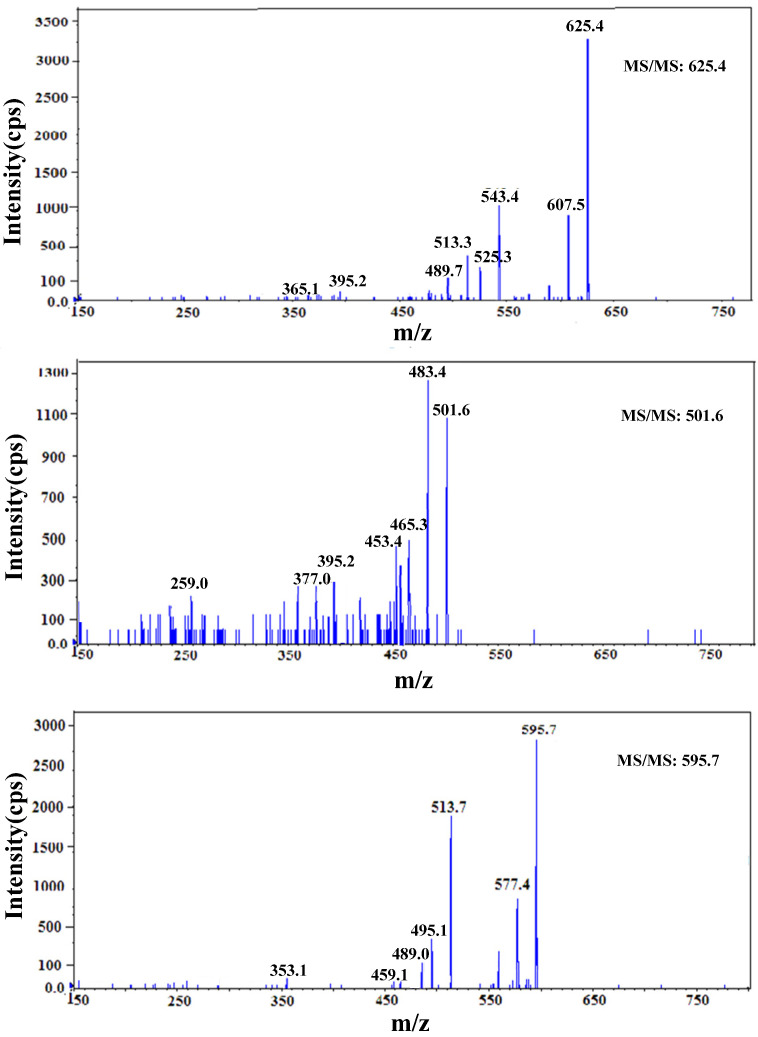
Secondary mass spectra of parent ions m/z 625.4, m/z 501.6 and m/z 595.7.

**Figure 6:**
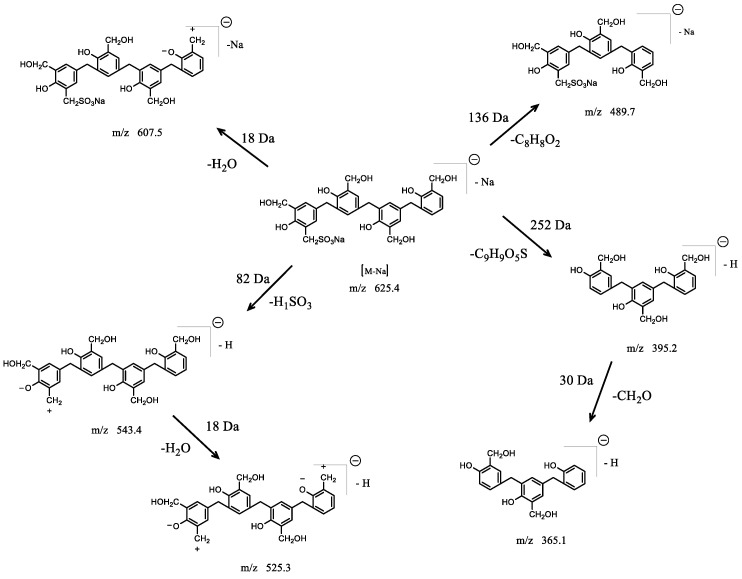
Proposed fragmentation pathway for the molecular ion with mass-to-charge ratio of m/z 625.4.

In order to further characterize the SPF resin structure, the molecular ions at m/z 625.4, 501.7, and 595.8 were analyzed with the MS/MS spectra, as shown in Figure 5. The MS/MS spectrum of the ion at m/z 625.4 generated many product ions at m/z 607.5, 543.4, 525.3, 513.3, 489.8, 395.2, and 365.1. According to the FTIR results, the phenol rings were connected by methylene bridges in the position of o–p′. The proposed breakdown scheme of the molecular ion of m/z 625.4 is shown in Figure 6. The presence of m/z 607.5, 543.4, 489.8, and 395.2 ions arose from the loss of H_2_O (18 Da), H_1_SO_3_ (82 Da), C_8_H_9_O_2_ (137 Da), and C_9_H_10_O_5_S (253 Da), respectively. Further fragmentations were produced when H_2_O (18 Da) and CH_2_O (30 Da) were lost from the ions at m/z 543.4 and 395.2 to form m/z 525.3 and 365.1, respectively.

**Figure 7:**
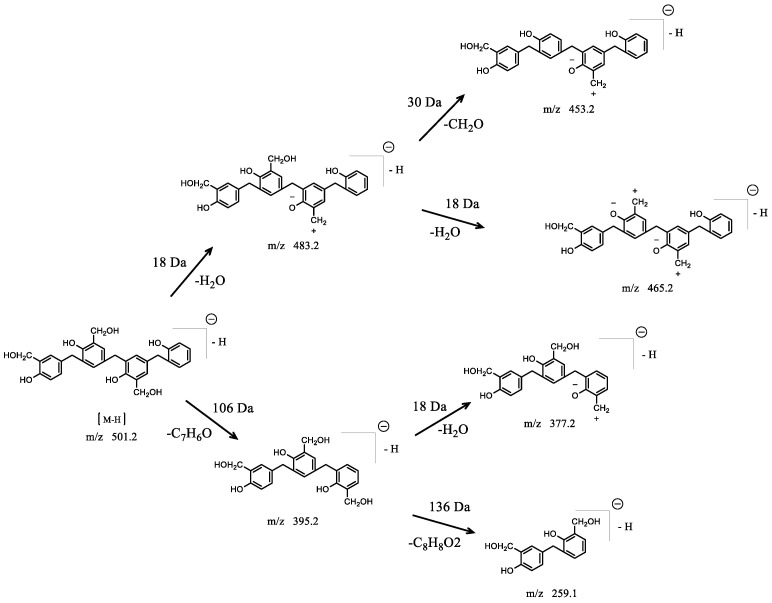
Proposed fragmentation pathway for the molecular ion with mass-to-charge ratio of m/z 501.2.

The MS/MS spectrum of the ion at m/z 501.6 produced six product ions of m/z 483.4, 465.3, 453.4, 395.2, 377.0, and 259.0. The proposed fragmentation pattern for the ion at m/z 501.7 is displayed in Figure 7. The most abundant ion at m/z 483.4 of composition C_30_H_26_O_5_- was generated from the loss of H_2_O (18 Da). The loss of a second H_2_O (18 Da) and the loss of hydroxymethyl (30 Da) produced the product ions at m/z 465.3 and 453.4, respectively. The produced ion at m/z 395.2 originated from the consecutive loss of methylene and phenol (106 Da). This suggested that there was no hydroxymethyl group on the end phenol ring. The ion at m/z 377.0 indicated the loss of H_2_O (18 Da) from the ion at m/z 395.2. Alternatively, the ion at m/z 259.0 resulted from the loss of C_8_H_8_O_2_ (136 Da) from the ion at m/z 395.2, which is a typical fragmentation for phenol–formaldehyde resin.

**Figure 8:**
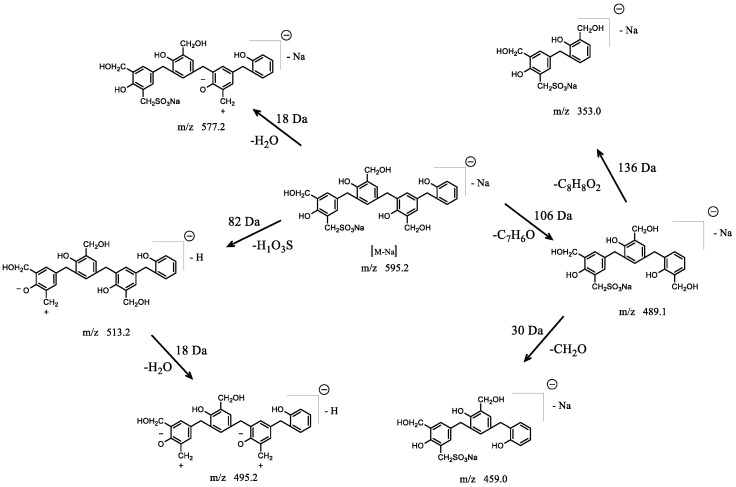
Proposed fragmentation pathway for the molecular ion with mass-to-charge ratio of m/z 595.2.

Figure 8 illustrates the proposed fragmentation pathway for the molecular ion at m/z 595.7, which produced multiple product ions at m/z 577.4, 513.7, 495.1, 489.0, 459.1, and 353.1. A loss of H_2_O (18 Da) resulted in the intensive ion at m/z 577.4. The ions at m/z 513.7 and 495.1 could correspond to the loss of H_1_O_3_S (82 Da) and the consequent losses of H_2_O_3_S and H_2_O (82+18 Da), respectively. The presence of product ions at m/z 489.0 and 353.1 indicated the losses of one and two phenol units from the molecular ion at m/z 595.7. The ion at m/z 489.0 was related to the loss of C_7_H_6_O (106 Da), which indicated that no hydroxymethyl group was on the end phenol unit. Further losses of ions of m/z C_8_H_8_O_2_ (136 Da) and CH_2_O (30 Da) from m/z 489.0 resulted in the ions at m/z 353.1 and 459.1, respectively.

#### 3.2.3. Effect of Formaldehyde/Phenol Molar Ratio(F/P) on SPF Resin Properties

**Figure 9:**
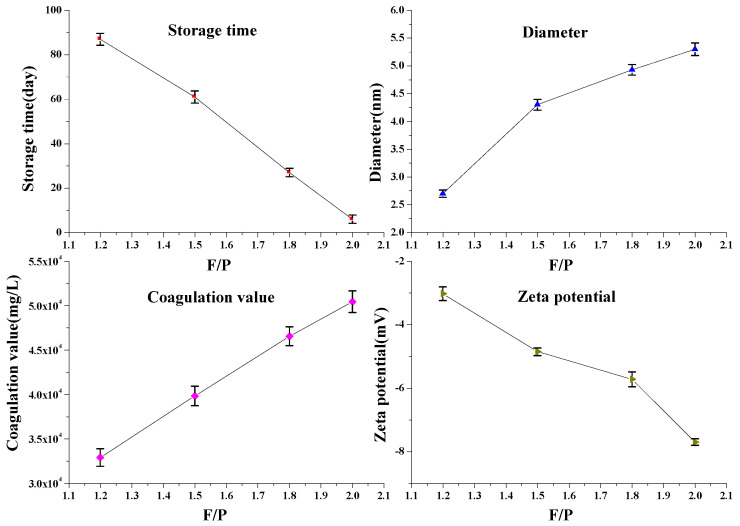
Effects of F/P on the SPF resin properties (mean ± s.d., *n* = 3 per condition).

Formaldehyde introduces a hydroxymethyl group on the phenol via an additive reaction. The hydroxymethyl group reacts with the active site of phenol to undergo polycondensation. Therefore, F/P has a greater effect on the storage stability of SPF. In addition, the salt tolerance of the SPF resin is also an important parameter affecting the application of SPF resin. The properties of SPF resin are expected to be influenced by the F/P molar ratio. The effects of F/P on the SPF resin performance (storage time, coagulation value, average diameter, and zeta potential) are shown in Figure 9. It is observed that when the P/F increased from 1.2 to 2.0, the storage time decreased from 86 to 6 days; however, when the P/F was 2.2, the SPF was completely solidified during the preparation. The coagulation value and average diameter all increased as the F/P molar ratio increased. The zeta potential of SPF was negative due to the ionization of phenolic hydroxyl groups, and the zeta potential decreased from −3.02 to −7.70 mV with the increase in P/F. The increase in the average diameter of the SPF resin with F/P was attributed to the fact that the increase in F/P can increase the additive reaction of formaldehyde to obtain more hydroxymethyl groups, which results in the acceleration of condensation reaction, thus leading to a greater diameter of SPF resin molecules. Accordingly, more hydroxymethyl groups on the aromatic rings with the increased F/P can also promote the condensation reaction during the storage, which results in decreased storage time. The increase in the absolute value of the zeta potential with the increase in F/P was mainly attributed to the molecular size. An increase in molecular size leads to more charge on the molecular structure, as reported by Andersen [24]. Accordingly, the coagulation value increased from 3.291 × 10^4^ to 5.045 × 10^4^ mg/L due to more surface charge on the SPF molecular structure. The above results indicated that, although the storage time of SPF could be prolonged by reducing the P/F, it would simultaneously reduce the number of hydroxymethyl groups on the phenolic resin, which would cause a decrease in the gelation properties. Therefore, the storage stability and gelling properties should be comprehensively evaluated when using SPF as a cross-linker.

#### 3.2.4. Effects of Sodium Formaldehyde Sulfoxylate/Phenol Molar Ratio (S/P) on PFR Performance

**Figure 10:**
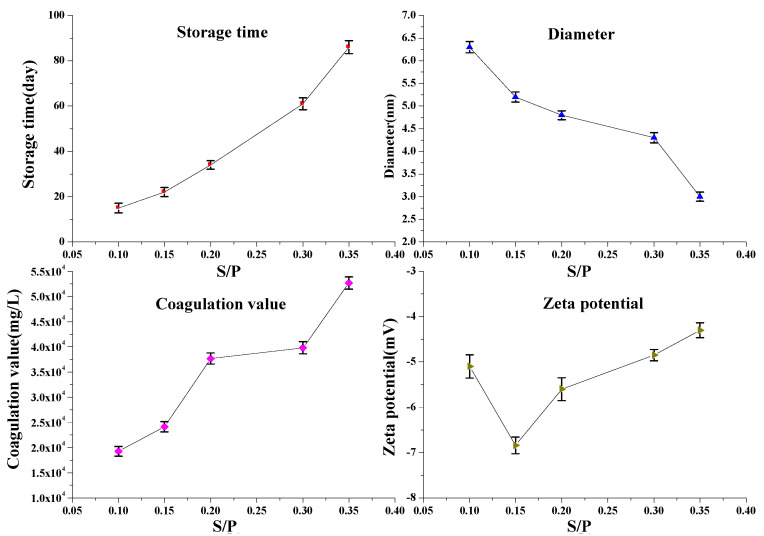
Effects of S/P on the SPF resin properties (mean ± s.d., *n* = 3 per condition).

Figure 10 shows the effects of S/P on the storage time, coagulation value, average molecular diameter, and zeta potential of SPF resin molecules. As expected, the average diameter decreased with the increase of S/P, while the storage time increased with the increase of S/P. An increase in S/P can form more sulfonate groups on the molecular structure, which occupies the activity sites on the phenolic rings mainly in either the ortho- or para- position. This can restrain the condensation reaction, resulting in a decrease in diameter and an increase in storage time. However, the zeta potential exhibited complicated dependence on the S/P. The absolute value of the zeta potential first increased to the maximum and then decreased with the S/P. This phenomenon can be explained by the fact that the zeta potential is related to the average diameter and average surface charge density. The absolute value of the zeta potential increased with an increase in the average diameter, as shown in Figure 9. On the other hand, the absolute value of zeta potential also increased with the increase in S/P. Ultimately, the absolute value of zeta potential first increased with the S/P (<0.15)and then decreased with the S/P (>0.15). Figure 10 also shows that the coagulation value increased with the increased S/P, indicating that an increase in the sodium formaldehyde sulfoxylate concentration can effectively increase the salt resistance for SPF resin. This phenomenon is mainly ascribed to the fact that the increase in S/P can increase the sulfonic groups on SPF resins, resulting in higher repulsive energy between two SPF resin molecules and thus leading to a higher coagulation value with the increased S/P.

**Figure 11:**
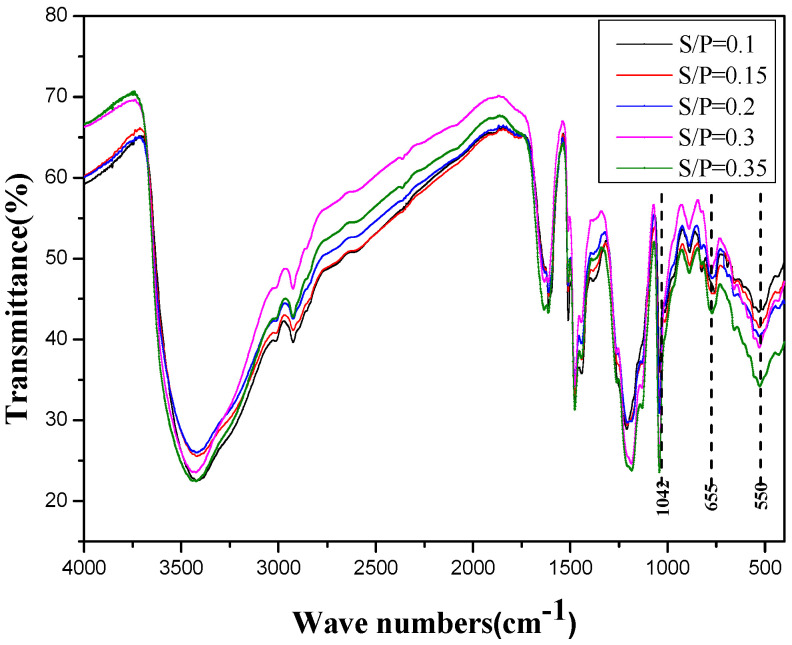
FTIR spectra of SPF resins with different S/P in the range of 0.1–0.35.

In order to investigate the structural changes that occurred between the samples with different S/P values, the FTIR spectra of the samples obtained after freeze-drying under vacuum were recorded, as shown in Figure 11. The FTIR spectra revealed that there were obvious differences in the adsorptions at 1042, 655, and 550 cm^−1^. The adsorption at 1042 cm^−1^ was a characteristic absorption value for a sulfonic group. The adsorption at 655 cm^−1^ was assigned to the C-S stretching vibration, and the adsorption at 650 cm^−1^ was mainly attributed to the S-O stretching vibration of a sulfonic group. It is clear that the adsorption values at 1042, 655, and 550 cm^−1^ all increased with the S/P, indicating that the SPF resin can be successfully sulfonated by sodium formaldehyde sulfoxylate. 

## 4. Discussion

When phenolic resin is used as a cross-linker of polyacrylamide profile control and as a water-plugging agent, it easily aggregates at high ionic strength and is prone to polymerization reaction and even solidification during storage. All these factors have great influences on the field application effect of PF resin. The ortho–para position of the phenolic hydroxyl group on phenol has three HOMO active reaction sites, which can react with formaldehyde to produce a hydroxymethyl group. The hydroxylmethyl group can react with the amino group of the polyacrylamide to form a gel compound. However, reactions can also occur between the hydroxylmethyl groups and between the hydroxylmethyl group and the active site of phenol, so that both additive and condensation polymerization reactions occur in the reaction system of phenol and formaldehyde. The condensation polymerization reaction is the dehydration reaction between the hydroxymethyl group and the phenol active site, which results in a reduction in the water solubility of PF resin after polycondensation. Sodium formaldehyde sulfoxylate was added to the phenol–formaldehyde system, which reacts with the phenol active site to produce sulfoxylate. This functional group can significantly increase the water solubility of the phenolic resin, and at the same time, the active site of the phenol occupying polycondensation decreases, which reduces the degree of polycondensation reaction and increases its storage stability. At the same time, it does not affect the reaction between the hydroxymethyl group and the amino group of polyacrylamide, so the phenolic resin retains its ability to cross-link with polyacrylamide.

## 5. Conclusions

A novel sulfonated phenol–formaldehyde resin was synthesized under basic conditions. The reaction process of SPF resin was conducted while measuring the viscosity of the resin solution, which indicated that the presence of sodium formaldehyde sulfoxylate can constrain condensation reaction. FTIR and HPLC–MS/MS were used to characterize the molecular structure of the SPF resin. The result showed that the SPF resin was successfully sulfonated by the -CH_2_SO_3_Na group, and phenol rings were connected by methylene bridges in the position of o–p′. The SPF resin molecules were not all sulfonated by sulfonate groups when S/P was 0.2, and the sulfonated ones only had one sulfonate group on the chain ends of the polymer molecules. The F/P and S/P ratios were found to have great influences on the properties of SPF resin. When increasing the F/P from 1.2 to 2.2, the molecular diameter increased with the increase in F/P, while the storage time decreased with the increased F/P due to more hydroxymethyl groups on SPF resin samples with the increased F/P. Accordingly, the coagulation value and the absolute value increased with the increase in F/P due to greater negative zeta potential with higher molecular diameter. An increase in S/P resulted in more sulfonate groups on the SPF resins, which increased the storage time and coagulation value. The molecular diameter decreased with the S/P increase due to fewer active sites that were occupied by sulfonate groups. However, the zeta potential first decreased and then increased with the increased S/P because of its complex dependence on the molecular size and sulfonation degree.

## Figures and Tables

**Table 1:** Mass spectra of SPF resin in different retention times.

Retention Time	Exptl	[M-H]^−^	[M-Na]^−^	P	H	S
		cacd	cacd			
21–26 min	319.5	320.3	-	2	4	0
	425.6	426.4	-	3	4	0
	455.4	456.4	-	3	5	0
	531.6	532.5	-	4	3	0
	561.5	562.6	-	4	5	0
	591.5	592.6	-	4	6	0
31.7–32.6 min	459.5	-	459.5	3	2	1
	489.5	-	489.5	3	3	1
	595.5	-	595.6	4	3	1
	625.5	-	625.6	4	4	1
	655.6	-	655.6	4	5	1
	761.9	-	761.7	5	5	1
	791.6	-	791.8	5	6	1
	927.7	-	927.9	6	7	1
	1063.7	-	1064.0	7	8	1
34.7–35.0 min	365.6	366.4	-	3	2	0
	459.6		459.5	3	2	1
	501.7	502.5	-	4	3	0
	595.8	-	595.6	4	3	1
	638.0	638.7	-	5	4	0
	701.3	-	701.7	5	3	1
	731.8	-	731.7	5	4	1
	773.9	774.8	-	6	5	0
	837.7	-	837.9	6	4	1
	867.9	-	867.9	6	5	1
	973.8	-	974.0	7	5	1
	1003.8	-	1004.0	7	6	1
	1033.9	-	1034.0	7	7	1
	1040	-	1140.1	8	7	1
	1276	-	1276.3	9	8	1
	1306	-	1306.3	9	9	1

Exptl is experimental data. P, H, and S denote the number of phenol rings and hydroxymethyl and methanesulfonic groups in the copolymer structure, respectively.

## Data Availability

All data generated or analyzed during this study are included in this article.
